# Tracking evolving communities in fake news cascades using temporal graphs

**DOI:** 10.1038/s41598-026-35175-4

**Published:** 2026-01-09

**Authors:** Yanfei Ma, Daozheng Qu, Yibo Wang

**Affiliations:** 1 Department of Computer Science, Fairleigh Dickinson University, Vancouver, V6B 2P6 Canada; 2 Department of Computer Science, University of Liverpool, Liverpool, L69 3DR UK; 3 Rady School of Management, University of California San Diego, San Diego, 92093 USA

**Keywords:** Dynamic community detection, Fake news cascades, Temporal graph networks, Reinforcement learning, Social media fake news, Mathematics and computing, Physics

## Abstract

Misinformation proliferates on social media platforms owing to both static and dynamic user populations, where the set of active users and their interactions evolve over time. The development, amalgamation, or disintegration of communities throughout an information cascade complicates the longitudinal tracking of these communities. Numerous contemporary methodologies either neglect temporal factors or employ static clustering techniques, which do not accommodate dynamic coordination. We propose TIDE-MARK, a methodology designed to identify communities inside fake news cascades that exhibit consistency in both structure and temporal dynamics. The methodology encompasses node embeddings via temporal graph neural networks, prototype-driven clustering, Markov modeling of community transitions, and reinforcement-based refinement. The unified design facilitates consistent and comprehensible community trajectories. Three empirical datasets pertaining to political, entertainment, and health-related fake news are utilized to evaluate TIDE-MARK. The databases include PolitiFact, GossipCop, and ReCOVery. Our model surpasses robust baselines regarding structural (modularity, conductance) and temporal (adjusted Rand index) measures, supported by consistent effect sizes. Structural research indicates that real news spreads through more scattered and less organized communities, while false news propagates through more stable and well interconnected communities. Our objective is to assess the viability of interventions by simulating a structure-aware approach that targets important users in nascent communities. The substantial reduction in cascade modularity and spread demonstrated in the results demonstrates the potential viability of content-neutral mitigation techniques. TIDE-MARK offers a structure-aware framework for real-time fake news monitoring, emphasizing network-based strategy signals over textual analysis. It establishes a foundation for innovative methods of dynamic community monitoring inside complex social systems and features an interpretable architecture that enables ethical application.

## Introduction

In today’s information landscape, misinformation spreads rapidly across social media^1^, affecting public opinion, electoral outcomes, and public health decision-making^2^. This spread typically originates from coordinated user communities that consistently reinforce false or misleading messages, rather than from solitary individuals. Comprehending the origin, evolution, and maintenance of influence within these communities is essential for the early detection and effective abatement of disinformation. This real-world imperative drives our investigation of dynamic community growth amid misinformation cascades.

Understanding how user communities emerge and evolve over time is central to analyzing collective behavior in dynamic social systems. On social media platforms, interactions are not static but unfold through ongoing conversations, content sharing, and structural adaptation. This dynamic nature introduces challenges for detecting communities whose composition and connectivity evolve across time^3,4^.

Beyond community-level analysis, several works directly model fake news propagation as cascades. For example, Ma et al.^5^ proposed a sequential model that captures temporal diffusion patterns for rumor detection. However, such approaches emphasize early classification of cascades rather than the structural evolution of communities, leaving the role of persistent clusters in misinformation spread underexplored. In the context of misinformation, these challenges become more pronounced. Studies have shown that fake news campaigns often exploit *persistent communities*–densely connected user clusters that remain stable across time–to amplify false narratives^1,6–8^. These communities differ from organically formed groups in real news cascades; they are temporally synchronized, topologically tight, and behaviorally aligned^9^. Their persistence makes them ideal vehicles for repeated exposure and coordinated manipulation. Detecting such structures is thus essential for timely intervention and early warning.

Beyond misinformation, Juul and Ugander^10^ systematically analyzed structural differences among information diffusion mechanisms, demonstrating that cascades with similar sizes can exhibit distinct topological signatures. This insight reinforces the importance of structural modeling for distinguishing coordinated propagation from organic diffusion, which motivates the design of TIDE-MARK. These observations highlight the need to situate dynamic community detection within the broader literature on misinformation diffusion.

At the same time, recent stance detection studies challenge the assumption that GNNs always dominate. Pick et al.^11^ proposed STEM, a structural embedding method that outperformed GNNs under domain shifts. Similarly, Barel et al.^12^ introduced Acquired TASTE, which integrates textual and structural features, showing that classical spectral methods can compete with or surpass deep GNNs. These findings underscore the importance of modularity and interpretability–qualities that TIDE-MARK explicitly integrates by decoupling embeddings, temporal forecasting, and reinforcement-guided refinement.

To address the above gaps, we introduce TIDE-MARK, a modular and interpretable framework for tracking dynamic communities in fake news cascades. It integrates three synergistic components: (1) temporal GNN embeddings that capture evolving user behavior, (2) Markov modeling to forecast transitions in community assignments, and (3) reinforcement learning to refine boundary nodes via structure- and time-sensitive rewards. Unlike monolithic approaches, this architecture separates representation, dynamics, and decision policy, ensuring flexibility and interpretability across datasets.

We evaluate TIDE-MARK on three well-established datasets–PolitiFact, GossipCop, and ReCOVery–covering misinformation across political, entertainment, and health domains^13,14^. Our experiments assess structural stability, temporal coherence, and the feasibility of topology-aware interventions for mitigating misinformation spread.

Each component directly targets a certain gap already identified. The temporal GNN embeddings address the instability of snapshot-based clustering by incorporating dynamic user interactions into time-sensitive node representations. The Markov transition module alleviates temporal inconsistency by explicitly modeling the evolution of communities across snapshots, hence assuring seamless yet adaptable membership changes. Ultimately, the reinforcement-based refinement addresses remaining boundary ambiguity by optimizing structure- and time-sensitive rewards, resulting in coherent and interpretable community bounds. Collectively, these components address the methodological deficiencies among static modularity optimization, temporal embedding, and interpretable community tracking.

In addition to these empirical assessments, TIDE-MARK also enhances the conceptual understanding of the interaction between temporal and structural dynamics in developing social networks. TIDE-MARK distinctly separates representation, transition, and refinement into interpretable steps, in contrast to other pipelines that conflate embedding and clustering. This modular framework reconceptualizes dynamic community detection as a temporally consistent decision-making process, illustrating the coexistence of stability and adaptation in collective behavior. The concept provides a theoretical perspective on the emergence and sustained influence of persistent clusters in misinformation cascades by modeling community evolution through Markov-consistent transitions and reinforcement-driven feedback.

## Related work

### Dynamic community detection paradigms

Dynamic community detection methods can be broadly grouped into four paradigms. *Snapshot-based clustering* applies static community detection (e.g., Louvain^15^, Infomap^16^) independently on each temporal slice, which is efficient but may produce fragmented trajectories across time^17,18^. *Latent probabilistic models* (e.g., hidden Markov models^19^ and dynamic stochastic block models^20^) introduce explicit temporal dependencies but can be difficult to scale and may underutilize fine-grained topology. *Temporal graph neural networks* learn time-aware node representations^21,22^ and have shown strong performance in temporal prediction, yet community tracking pipelines built on embeddings can remain sensitive to noise and often lack explicit mechanisms for temporal stability. More recently, *streaming community detection* maintains communities online as interactions arrive, improving scalability and immediacy for monitoring scenarios^23^; however, temporal stability and interpretability remain challenging. We review these lines of work in more detail in the following subsections.

### Classical and evolutionary community detection

Community detection research has historically focused on structural partitioning, with foundational classical methods including modularity optimization^24^ and spectral clustering^25^. Nevertheless, these static methodologies sometimes neglect to account for temporal evolution, leading to inconsistent partitions in dynamic contexts. Subsequent studies implemented temporal smoothness requirements to improve cross-snapshot consistency in order to overcome this issue^3,26^. Evolutionary spectral clustering^27^ further formalized this principle by jointly optimizing current and historical cluster quality. These methodologies established the conceptual framework for dynamic community discovery but were deficient in scalability and interpretability within extensive social networks.

### Temporal graph neural networks

Recent advances in temporal graph neural networks (TGNNs) have enabled fine-grained modeling of node interactions over time. Methods such as DySAT^28^ and TGAT^29^ learn temporal embeddings through self-attention and inductive message passing, while TGN^22^ introduces memory mechanisms to capture evolving node states. While these models proficiently capture temporal connections, they predominantly focus on node-level prediction and link forecasting tasks. Conversely, TIDE-MARK utilizes temporal embeddings at the community level, integrating them with probabilistic modeling and reinforcement refinement to get interpretable temporal coherence.

### Contemporary deep dynamic graph models

Beyond classical TGNNs, several recent models explore deep dynamic graph learning. EvolveGCN^21^ updates GCN parameters recurrently to adapt to temporal changes, while HTNE^30^ models temporal neighborhood formation for event-driven networks. Recent studies have also combined graph convolution and contrastive learning to detect dynamic communities in sparse temporal networks^31^. We also regarded these contemporary architectures as possible baselines. Nonetheless, they are mostly intended for node-level functions rather than monitoring community evolution. Consequently, direct experimental comparison would be unsuitable owing to intrinsic differences in the tasks. Beyond TGNNs, recent graph-ML clustering advances on attributed/multiview graphs– e.g., link-based attributed graph clustering via approximate generative Bayesian learning^32^ and multiview fusion GNNs with fuzzy clustering^33^– offer complementary priors and representation strategies that are promising for community refinement.

### Information cascades and misinformation propagation

Alongside analytical advancements, disinformation research has highlighted that the dissemination of bogus news adheres to unique structural and temporal patterns. Vosoughi et al.^1^ and Shu et al.^13^ show that false information spreads faster and broader than factual news, often through cohesive and persistent communities. Other studies on cascade dynamics^34^ and cross-platform propagation^35^ highlight the importance of structural signals alongside textual analysis. Juul and Ugander^10^ further showed that cascades of similar size can differ substantially in structure, emphasizing the need to model diffusion mechanisms via their topological patterns. TIDE-MARK integrates interpretable reinforcement learning with temporal community detection, linking methodological innovation to practical misinformation analysis and structural intervention.

## Materials and methods

To ensure reproducibility and to ground our model in a realistic application scenario, we begin by describing the datasets and temporal graph construction process used in this study.

### Datasets

Three publicly available datasets are utilized to encompass a wide range of fake news types: the PolitiFact and GossipCop subsets of FakeNewsNet^13^, along with the ReCOVery dataset^14^. The datasets encompass the domains of politics, entertainment, and health news, respectively.

PolitiFact and GossipCop feature news articles that have undergone individual fact-checking and are categorized as either fake or real. ReCOVery assesses reliability at the source level, categorizing each news item as reliable or unreliable according to the credibility of its publisher. All datasets contain tweet identifiers that facilitate the reconstruction of user-level propagation cascades on Twitter.

To achieve equitable and uniform assessment, we select 30 fake or unreliable cascades from each of the three datasets–PolitiFact, GossipCop, and ReCOVery–resulting in a total of 90 cascades. Each cascade comprises a minimum of 100 distinct users and extends over at least five time intervals. This selection strategy guarantees both structural and temporal comparability among datasets, concentrating exclusively on the propagation of fake news.

The original datasets indicate that the complete FakeNewsNet (PolitiFact and GossipCop) and ReCOVery corpora encompass more than 9,000 news articles, 1 million users, and 4 million retweet or reply exchanges. To ensure a controlled and equitable assessment, we examine a representative subset of 30 cascades from each dataset, chosen using stratified random selection according to cascade size and temporal duration. Each experiment is conducted five times using distinct random seeds, and the findings are averaged over the trials to guarantee reproducibility and statistical robustness.

### Overview of the TIDE-MARK framework

This study aims to detect and track the evolution of user communities involved in the dissemination of fake news on social media. These communities may represent coordinated amplifiers, skeptical observers, or organically formed interest clusters, whose structure and persistence vary across the information cascade.

To address this, we propose **TIDE-MARK** (Temporally-Integrated Deep Embedding with Markov-Adaptive Reinforcement), a modular framework designed to produce temporally consistent community partitions across dynamic propagation networks.

**Fig. 1:**
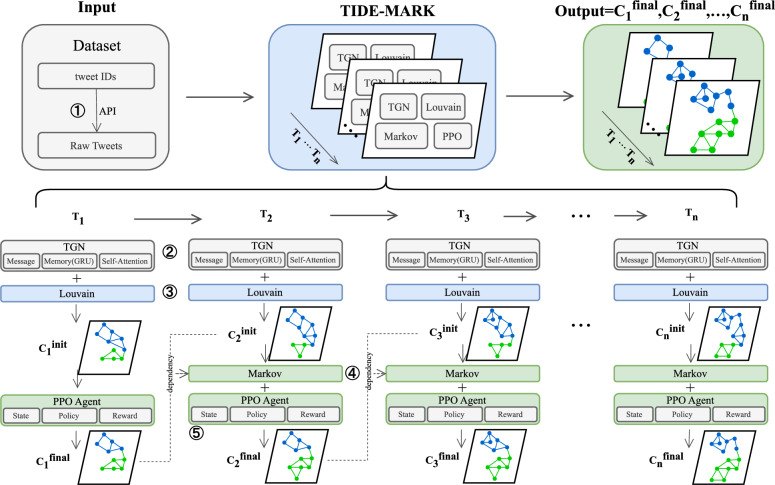
Overview of the **TIDE-MARK** framework. The framework consists of five stages: (1) Preprocessing, (2) Temporal Graph Embedding, (3) Initial clustering, (4) Markov transition forecasting, and (5) Reinforcement-based refinement.

The TIDE-MARK framework consists of five sequential stages as illustrated in Fig. 1:

1. **Preprocessing**: Raw tweet IDs are used to reconstruct misinformation cascades into a sequence of temporal graph snapshots $\{G_t\}$, where nodes represent users and edges reflect interaction (e.g., retweet, reply) events within discrete time intervals.
2. **Temporal graph embedding**: A Temporal Graph Network (TGN) learns node embeddings $\textbf{z}_v^t$ at each time *t*, encoding temporal context, structural position, and interaction dynamics.
3. **Prototype-guided initial clustering**: To obtain community assignments from embeddings, we construct a similarity graph by linking nodes based on cosine similarity in the embedding space (e.g., via mutual *k*-nearest neighbors). Louvain is then applied to this reconstructed similarity-weighted graph to yield initial community partitions. This approach preserves the modularity-based objective of Louvain while enabling it to operate in semantically meaningful latent spaces.
4. **Markov-based transition modeling**: A state transition model is built by tracking the evolution of community memberships across snapshots, computing empirical transition probabilities between community labels over time. This enables modeling of community drift, splits, and merges.
5. **Reinforcement-based refinement**: A Proximal Policy Optimization (PPO) agent iteratively refines community boundaries. The agent selects boundary nodes (connected to multiple communities) and reallocates them to maximize an objective that balances structural modularity and temporal alignment across frames.

This end-to-end pipeline produces community labels that are both structurally cohesive and temporally stable, enhancing our understanding of the coordinated spread of misinformation.

Given a dynamic graph sequence with optional node features $\textbf{X}_t$, the goal is to compute community partitions $\mathscr {C}_t$ that maintain structural coherence at each time step while ensuring temporal smoothness across steps. This design enables TIDE-MARK to support interpretable analysis of how user communities form, evolve, and interact in the context of news propagation.

#### Stage 1: preprocessing

Utilizing tweet identifiers from each article, we retrieve comprehensive tweet metadata via the Twitter API, encompassing tweet content, user details, timestamps, and interaction kinds, including retweets and replies. Every item, whether labeled as fake, real, reliable, or unreliable, generates a retweet cascade that illustrates the dissemination of information among users.

We depict each cascade as a dynamic interaction graph. Users are represented as nodes, with directed edges indicating retweet or reply activities between them. To capture temporal evolution, we partition each cascade into a series of fixed-length time intervals (e.g., one hour each snapshot), resulting in a dynamic graph $\mathscr {G} = \{G_1, G_2,..., G_T\}$, where each $G_t$ represents user interactions during the *t*-th interval.

Each snapshot of $\textbf{X}_t$ is generated by amalgamating two categories of information: (1) semantic tweet embeddings derived from a pre-trained BERT model^36^, and (2) user profile metadata, encompassing account creation date, follower count, and verification status. Timestamps are standardized within each window to improve temporal alignment and consistency in subsequent embedding and learning processes.

The semantic tweet embeddings and user profile metadata are amalgamated into a cohesive feature vector for each node, which functions as the input representation $\textbf{X}_t$ across all temporal snapshots. The Louvain clustering employed in TIDE-MARK adheres to the conventional undirected modularity framework, as user interactions (retweets and responses) are symmetrized to represent reciprocal connectedness strength.

This preprocessing architecture guarantees that both textual semantics and user behavioral features are represented cohesively, allowing the model to differentiate between content-driven dissemination and user coordination patterns characteristic of misinformation cascades.

#### Stage 2: temporal graph embedding

Given the node features $\textbf{X}_t$, we use the Temporal Graph Network (TGN)^22^ to generate evolving node embeddings. TGN provides memory-based embeddings that are especially suited for capturing asynchronous interactions, and has been shown to outperform earlier models like TGAT^29^ or DySAT^28^ in encoding fine-grained temporal dependencies.

Each node *v* maintains a memory-based hidden state $\textbf{h}_v^{(t)}$, from which a task-specific embedding $\textbf{z}_v^{(t)}$ is computed.

For each interaction (*u*, *v*) at time *t*, we construct a message:

1 $$\begin{aligned} \textbf{m}_{u \rightarrow v}^{(t)} = f_\text {msg}(\textbf{h}_u^{(t^-)}, \textbf{h}_v^{(t^-)}, \tau , \textbf{e}_{uv}^{(t)}), \end{aligned}$$

where $\tau$ denotes the elapsed time (in hours) since the last recorded interaction involving node *u* or *v*, and $\textbf{e}_{uv}^{(t)}$ represents optional edge attributes such as interaction type or timestamp.

Node memory is updated using a gated recurrent unit (GRU)^37^, enabling each node to aggregate temporal information from prior messages:

2 $$\begin{aligned} \textbf{h}_v^{(t)} = \textrm{GRU} \left( \textbf{h}_v^{(t^-)}, \sum _{u \in \mathscr {N}(v)} \textbf{m}_{u \rightarrow v}^{(t)} \right) . \end{aligned}$$

The final embedding $\textbf{z}_v^{(t)}$ is obtained via temporal self-attention over the *k* most similar neighbors:

3 $$\begin{aligned} \textbf{z}_v^{(t)} = \textrm{Attn}\left( \textbf{h}_v^{(t)}, \{\textbf{h}_u^{(t)} \mid u \in \mathscr {N}_k(v)\}\right) , \end{aligned}$$

where $\mathscr {N}_k(v)$ denotes the top-*k* neighbors of node *v* in similarity space. We empirically set $k = 20$ in all experiments to balance efficiency and context coverage.

In the misinformation context, these temporal embeddings capture how user influence and interaction patterns evolve, providing a dynamic representation of coordination intensity over time.

#### Stage 3: prototype-guided initial clustering

To derive an initial partition $C_t^{\text {init}}$, we construct a sparse similarity graph from embeddings $\textbf{z}_v^{(t)}$ by computing pairwise cosine similarity:

4 $$\begin{aligned} w_{uv}^{(t)} = \cos (\textbf{z}_u^{(t)}, \textbf{z}_v^{(t)}). \end{aligned}$$

Subsequently, we construct a *k*-nearest neighbor graph $\widetilde{G}_t$ by preserving the top-*k* neighbors for each vertex. This sparsification enhances clustering scalability and mitigates noise.

The Louvain algorithm^15^ is utilized on $\widetilde{G}_t$ to enhance modularity and discern coarse yet interpretable community structures, resulting in the initial assignment $C_t^{\text {init}}$.

Louvain is widely used for its optimization of modularity and interpretability. Although alternatives such as Infomap or Leiden are available, Louvain provides an favorable balance between accuracy and computational efficiency in dynamic clustering frameworks^15,38^.

This step provides coarse yet interpretable community partitions, serving as a structural baseline for analyzing coordinated diffusion groups in fake-news cascades.

#### Stage 4: Markov transition forecasting

To ensure temporal consistency, we model community transitions as a first-order Markov process^39^. Let $S_v^{(t)} \in \{1, \dots , k(t)\}$ denote the community label of node *v* at time *t*. For each pair of communities (*j*, *k*), we estimate transition probabilities:

5 $$\begin{aligned} p_{jk}^{(t)} = \frac{ \sum _v \textbf{1}[S_v^{(t-1)} = j \wedge S_v^{(t)} = k] + \lambda }{ \sum _{k'} \sum _v \textbf{1}[S_v^{(t-1)} = j \wedge S_v^{(t)} = k'] + \lambda \cdot k(t) }, \end{aligned}$$

where $\lambda = 0.1$ is the Laplace smoothing factor, and *k*(*t*) is the number of communities.

This results in a transition matrix $\textbf{P}^{(t)} \in \mathbb {R}^{k(t-1) \times k(t)}$, which captures node migration patterns and informs refinement decisions in the next stage.

By modeling community evolution as a Markov process, the framework captures the gradual continuity of user coordination rather than abrupt structural shifts, which is often observed in misinformation diffusion.

#### Stage 5: reinforcement-based refinement

We define boundary nodes as those connected to at least one neighbor in a different community, i.e., nodes *v* with $\exists u \in \mathscr {N}(v)$ such that $C_t(v) \ne C_t(u)$.

We formulate the reassignment of boundary nodes as a Markov Decision Process (MDP). Each state *s* is defined as:

6 $$\begin{aligned} s = \big (v,\, c,\, \textbf{z}_v^{(t)},\, \textbf{P}_{c,*}^{(t)}\big ), \end{aligned}$$

encoding the current node embedding, assigned community, and the corresponding transition vector.

The action space $\mathscr {A}(s)$ consists of “stay” and transfers to alternative communities. Each action is evaluated via the reward:

7 $$\begin{aligned} r = \Delta Q + \alpha \cdot \Delta \!\operatorname {Cond} + \beta \cdot p_{c \rightarrow cx2019;}^{(t)}, \end{aligned}$$

In this context, $\Delta Q$ denotes the modularity gain, while $\Delta \!\operatorname {Cond}$ signifies the change in conductance. The term $p_{c \rightarrow cx2019;}^{(t)}$ promotes transitions that are consistent with historical trends.

Specifically, $\Delta \!\operatorname {Cond}$ represents the variation in conductance prior to and following the reassignment of node *v* to community $c'$, indicating the distinctness of community boundaries.

**Avoiding metric circularity.** Given that modularity (*Q*) and conductance ($\Phi$) are utilized as evaluation metrics, we explicitly incorporate an extra *hold-out* criterion–**Normalized Mutual Information (NMI)**–to evaluate the agent’s generalization. NMI quantifies the mutual information between successive community partitions without incorporating the reward. Throughout training, the PPO agent is oblivious to NMI; this metric is calculated solely in retrospect for assessment, confirming that the policy enhances temporal coherence rather than simply manipulating the reward components. We preserve *Q* and $\Phi$ in the reward as they represent interpretable structural objectives, while the independent NMI metric protects against evaluation bias and circular optimization.

We train a Proximal Policy Optimization (PPO) agent to determine optimal actions. The training persists until the average modularity improvement $\Delta Q$ decreases below $10^{-3}$. The hyperparameters $\alpha$ and $\beta$ are tuned empirically.

This reinforcement refinement process incrementally modifies community borders in reaction to evolving diffusion patterns, which corresponds with the sporadic and adaptive characteristics of disinformation dissemination on social media.

### Algorithm summary

Algorithm 1 presents a pseudocode overview of the TIDE-MARK pipeline, demonstrating the construction and refinement of user communities through a series of temporal graph snapshots. The previous sections outline each phase of the framework, while this pseudocode elucidates the data flow, iterative control, and interdependencies among components.

At each time step *t*, the algorithm analyzes a dynamic user interaction graph $G_t$ by embedding nodes using TGN, clustering with the Louvain method, estimating temporal transitions, and refining boundary assignments through reinforcement learning. Boundary nodes are updated using a Proximal Policy Optimization (PPO) agent that employs a multi-factor reward system, which balances structural quality with temporal consistency.

The final output $\{\mathscr {C}_t\}_{t=1}^{T}$ consists of a temporally coherent sequence of community partitions, prepared for evaluation or visualization. The modular structure of the pseudocode illustrates the design of the framework, enhancing implementation and reproducibility.

**Algorithm 1:**
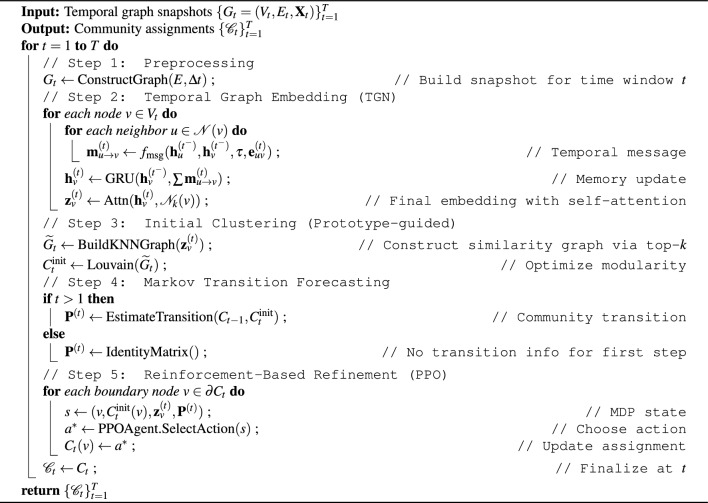
TIDE-MARK framework for tracking evolving communities. The algorithm processes a sequence of retweet graph snapshots to detect and refine user communities over time.

### Complexity analysis

This analysis focuses on the time complexity of each stage in the TIDE-MARK framework to evaluate its scalability. Let $|V_t|$ represent the number of nodes and $|E_t|$ represent the number of edges in the *t*-th snapshot. Let $k$ denote the top-$k$ neighborhood size utilized in attention and similarity construction, $B$ represent the number of boundary nodes taken into account for refinement, and $K$ indicate the number of candidate communities at each time step.

In Stage 1 (Preprocessing), the complexity of partitioning the edge stream into temporal windows is *O*(|*E*|) for the entire cascade. Feature extraction, which encompasses BERT-based semantic embedding and user metadata hydration, functions across all nodes, resulting in a computational cost of *O*(|*V*|), and is amenable to parallelization.

Stage 2 (Temporal Graph Embedding) constitutes the primary source of computational expense. Each interaction edge generates a message that updates node memory through a gated recurrent unit (GRU). The final embeddings are derived by applying attention mechanisms to the top-*k* neighbors for each node, yielding a total complexity of $O(|E_t| + |V_t| \cdot k)$ for each snapshot. Sparse attention guarantees that $k \ll |V_t|$.

In Stage 3 (Prototype-Guided Initial Clustering), the construction of a *k*-nearest neighbor graph from the learned embeddings necessitates $O(|V_t| \cdot k)$ operations. The application of the Louvain algorithm to this graph results in a practical cost of $O(|E_t| + |V_t| \log |V_t|)$, utilizing modularity heuristics that exhibit near-linear scalability in practice.

Stage 4 (Markov Transition Forecasting) estimates a $K \times K$ community transition matrix by analyzing node-level label changes across consecutive snapshots. This procedure necessitates $O(|V_t| + K^2)$ operations, with the latter term representing the computation of Laplace-smoothed transition counts. Given that $K \ll |V_t|$ in typical scenarios, this step is computationally efficient.

Stage 5 (Reinforcement-Based Refinement) involves the reassignment of boundary nodes through the resolution of a Markov Decision Process utilizing a Proximal Policy Optimization (PPO) agent. Each boundary node, denoted as *B*, possesses an action space of size *O*(*K*), with each action requiring a lightweight inference via the PPO policy network. The complexity is $O(B \cdot K \cdot C_{\text {ppo}})$, with $C_{\text {ppo}}$ representing the per-action inference cost, which is generally 1–2 forward passes. This stage exhibits inherent parallelizability and advantages from sparse transitions. The overall time complexity of TIDE-MARK is given by:

8 $$\begin{aligned} O\!\left( T \cdot \Big [ |E_t| + |V_t| \cdot k + |V_t| \log |V_t| + K^2 + B \cdot K \cdot C_{\text {ppo}} \Big ] \right) , \end{aligned}$$

where *T* represents the number of snapshots, and $|V_t|$ and $|E_t|$ indicate the average number of nodes and edges per snapshot, respectively.

Real-world propagation graphs are generally sparse ($|E_t| = O(|V_t|)$), and given that both *k* and *K* are small constants, the overall complexity scales quasi-linearly with the input size. This validates the computational feasibility of our approach for large-scale fake news cascades.

### Implementation details

All temporal snapshots are generated using a fixed window of $\Delta t = 1$ hour, in accordance with the aforementioned preprocessing procedure.

All implementations utilize PyTorch 2.2 and PyTorch Geometric Temporal. Experiments are performed using a single NVIDIA RTX 3090 GPU, which has 24 GB of memory. In the context of TIDE-MARK, we established $k = 20$ for both the temporal self-attention mechanism and the construction of the similarity graph. The PPO agent undergoes training for 100 episodes utilizing the Adam optimizer, set at a learning rate of $10^{-3}$. Training ceases when the average modularity gain $\Delta Q$ drops below $10^{-3}$. Automatic Mixed Precision (AMP) facilitates mixed-precision training, enhancing computational efficiency. For clarity, NMI is never used in the reward, optimizer updates, or stopping criteria. It is computed only after training to validate that the learned policy generalizes beyond the optimized structural terms (*Q* and $\Phi$).

**PPO agent configuration.** The PPO agent follows an actor–critic architecture with two hidden layers (128 and 64 units) and ReLU activations. The discount factor is $\gamma = 0.95$, the clipping parameter $\epsilon = 0.2$, and the entropy coefficient is 0.01. A batch size of 64 is used for each update, and the value network shares parameters with the policy head except for the final layer. These settings were empirically selected for stable convergence and are consistent across all experiments reported in this paper.

**Cascade sampling and randomization.** Thirty cascades are chosen for evaluation from each dataset. Cascades with fewer than 100 users or fewer than five temporal snapshots are excluded. Cascades are selected from the remaining candidates by stratified random sampling, considering cascade size and temporal span to ensure variation in diffusion depth and length. All stochastic procedures utilize a constant seed (42) to ensure determinism. Each experiment is conducted five times using randomized node permutations, and the given findings reflect the mean values from these iterations. These approaches promote reproducibility and guarantee consistency among datasets. To guarantee equity between instances of fake and real news, sampling is conducted independently inside each category (fake vs. real) employing an identical stratified methodology. We further validated stability by conducting the complete sampling procedure with five different random seeds, noting minimal variance ($<0.02$ in modularity and conductance) among the iterations.

### Evaluation metrics

To evaluate both the structural integrity and temporal consistency of discovered communities in fake news cascades, we adopt the following five metrics:

**Modularity **(*Q*) measures intra-community density relative to a null model, indicating structural cohesion.

**Conductance **($\Phi$) captures the sharpness of community boundaries by comparing inter-community edges to internal connectivity.

**Temporal adjusted rand index (ARI)** quantifies the consistency of community assignments between consecutive snapshots, reflecting temporal smoothness.

**Runtime (seconds)** reports the average time to process each snapshot, measuring computational efficiency.

**Effect size and confidence interval** evaluate the magnitude and reliability of differences in performance. Standardized effect sizes (e.g., Cohen’s *d*) and 95% confidence intervals for key metrics, including modularity and ARI, are reported, utilizing bootstrap resampling^40^ with 1,000 replicates to ensure robustness. The statistics facilitate clearer comparisons, particularly between fake and real cascades, as well as in ablation studies.

**Normalized mutual information (NMI; hold-out).** NMI measures the information-theoretic resemblance between successive partitions without exchanging parameters or objectives with the reward. We calculate $\textrm{NMI}(C_t, C_{t+1})$ for each consecutive pair of snapshots and present the mean (along with the 95% confidence interval) throughout $t=1,\dots ,T-1$ and cascades. NMI is exclusively utilized for post-hoc validation, not during training, early halting, or model selection, to avoid metric circularity.

### Baselines

To assess the effectiveness of TIDE-MARK, we compare it against three representative baselines:

**Static Louvain**^15^: Applies modularity optimization independently to each snapshot without temporal modeling. Serves as a simple, non-temporal baseline.

**TGN + Louvain**^22^: Uses Temporal Graph Networks to learn dynamic node embeddings, followed by Louvain clustering per snapshot. Captures time-aware embeddings but lacks refinement mechanisms.

**DySAT + Louvain**^28^: Employs stacked temporal self-attention layers to generate temporal embeddings, combined with Louvain clustering.

**Evolutionary Spectral Clustering (ESC)**^27^: A dynamic clustering system that explicitly guarantees temporal continuity by minimizing a weighted amalgamation of current snapshot quality and historical divergence from the preceding division. ESC directly represents inter-snapshot continuity without the need for retraining embeddings at each iteration.

**LabelRankT**^41^: A dynamic community discovery approach based on label propagation that incrementally changes node labels over time while maintaining temporal consistency through the retention of historical labels. LabelRankT offers an effective and replicable benchmark for assessing temporal coherence.

All baselines are implemented on the same snapshots and assessed under uniform preprocessing and feature extraction protocols to guarantee comparability. The identical modularity optimization technique is employed for Louvain-based pipelines (Static Louvain, TGN+Louvain, DySAT+Louvain, and EvolveGCN + Louvain). Conversely, ESC and LabelRankT utilize their inherent temporal smoothness or label-propagation methods, yet are implemented on the identical sequence of graph snapshots for equitable comparison.

Both ESC and LabelRankT include explicit temporal smoothness restrictions, facilitating a fair comparison with TIDE-MARK regarding temporal coherence and stability.

## Results

We initially outline our evaluation procedure and overarching conclusions. We evaluate structural quality (modularity *Q*, conductance $\Phi$), temporal smoothness (temporal ARI), and an independent hold-out criterion (NMI between consecutive partitions) that is not utilized in training or early halting across PolitiFact, GossipCop, and ReCOVery. We evaluate TIDE-MARK in comparison to Louvain-based methodologies (Static Louvain, TGN+Louvain, DySAT+Louvain) and dynamic community-detection benchmarks using explicit temporal regularization (Evolutionary Spectral Clustering, LabelRankT). TIDE-MARK typically achieves the highest or statistically comparable scores on *Q*, $\Phi$, and ARI, and frequently excels in the hold-out NMI, indicating balanced optimization.

### Fake vs. real news: structural differences in community evolution

**Fig. 2:**
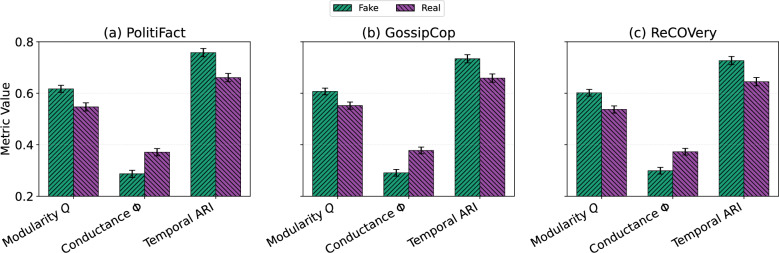
Structural differences between fake and real news across datasets. Each bar shows the average modularity, conductance, and temporal ARI for fake and real news cascades on PolitiFact, GossipCop, and ReCOVery. Fake news consistently exhibits higher modularity and temporal ARI but lower conductance, indicating tighter and more stable community evolution. Error bars represent 95% confidence intervals.

We evaluate the distinguishability of patterns in community evolution between fake and real news by comparing structural metrics–modularity (*Q*), conductance ($\Phi$), and temporal Adjusted Rand Index (ARI)–on matched cascades from three benchmark datasets: PolitiFact, GossipCop, and ReCOVery. For each dataset, we randomly sampled 30 fake and 30 real news cascades, each containing a minimum of 100 users, divided into five temporal snapshots. Metric values are averaged across time steps, cascades, and five random seeds. Confidence intervals (95% CI) are derived through bootstrap resampling, and effect sizes are expressed using Cohen’s *d*. Figure 2 summarizes these results across datasets, illustrating cross-domain differences in structural cohesion and persistence between fake and real news cascades.

**On PolitiFact**, fake news cascades show significantly higher modularity ($Q = 0.617$, CI: [0.603, 0.631]) than real news ($Q = 0.547$, CI: [0.531, 0.563]), with a large effect size ($d = 0.94$). Fake cascades also exhibit lower conductance ($\Phi = 0.315$, CI: [0.301, 0.329]) than real ones ($\Phi = 0.371$, CI: [0.357, 0.385]), $d = 0.71$, and higher temporal ARI (0.714 vs. 0.661, $d = 0.60$), indicating more persistent and internally cohesive community structures.

**On GossipCop**, the patterns are consistent: fake news has higher modularity ($Q = 0.611$, CI: [0.598, 0.624]) than real news ($Q = 0.552$, CI: [0.538, 0.566]), with $d = 0.91$; lower conductance ($\Phi = 0.323$ vs. 0.378, $d = 0.69$); and higher ARI (0.709 vs. 0.659, $d = 0.59$), further supporting the notion of fake news persisting within tightly knit communities.

**On ReCOVery**, fake news again demonstrates stronger structural cohesion and persistence: $Q = 0.599$ (CI: [0.586, 0.612]) vs. 0.537 (CI: [0.523, 0.551]), $d = 0.95$; $\Phi = 0.319$ (CI: [0.306, 0.332]) vs. 0.373 (CI: [0.360, 0.386]), $d = 0.68$; and ARI = 0.700 (CI: [0.684, 0.716]) vs. 0.645 (CI: [0.629, 0.661]), $d = 0.63$.

The findings indicate that fake news disseminates through more cohesive, persistent, and insular communities across all three datasets, whereas real news propagates in a more diffuse and decentralized manner. The structural distinctions offer a potential indicator for the early identification of fake news in content-agnostic contexts.

These gaps align with TIDE-MARK’s design: Markov-aligned transitions stabilize persistent clusters, while boundary refinement reduces fragmentation, yielding higher *Q*/ARI and lower $\Phi$ across time.

### Quantitative comparison with baselines

The performance of TIDE-MARK is evaluated on three benchmark datasets–PolitiFact, GossipCop, and ReCOVery–using five key metrics: modularity (*Q*), conductance ($\Phi$), temporal Adjusted Rand Index (ARI), hold-out Normalized Mutual Information (NMI) between consecutive partitions, and runtime per snapshot. Alongside temporal embedding-based approaches (TGN+Louvain and DySAT+Louvain), we also incorporate two dynamic baselines–Evolutionary Spectral Clustering (ESC) and LabelRankT–to facilitate a fair comparison with algorithms that explicitly describe temporal smoothness. NMI is calculated retrospectively for assessment purposes and is not utilized during training or reward optimization, functioning as an autonomous verification of metric circularity.

For each dataset, 30 unreliable cascades (each with at least 100 users) are selected, divided into five temporal snapshots, and assessed across five random seeds. Evaluation metrics are averaged over cascades and snapshots, with $95\%$ confidence intervals estimated by bootstrap resampling. Effect sizes (Cohen’s *d*) are reported to quantify practical significance.

**Fig. 3:**
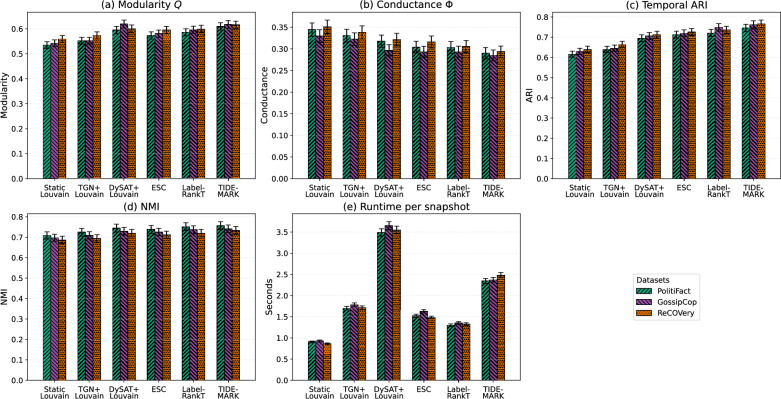
Structural, temporal, and efficiency performance across datasets. Each group of bars represents a method, with colors denoting PolitiFact (green), GossipCop (blue), and ReCOVery (orange). Subplots report: (**a**) Modularity *Q* (higher is better), (**b**) conductance $\Phi$ (lower is better), (**c**) Temporal Adjusted Rand Index (ARI; higher is better), (**d**) Hold-out NMI between consecutive snapshots (higher indicates temporal consistency), and (**e**) average runtime per snapshot (lower is better). Each bar aggregates results over 30 cascades $\times$ 5 snapshots $\times$ 5 runs. Error bars show $95\%$ confidence intervals. Cohen’s *d* effect sizes indicate the magnitude of improvement over the strongest baseline.

Figure 3 shows that TIDE-MARK consistently achieves top or near-top performance across datasets and metrics, balancing structural, temporal, and computational aspects. On PolitiFact, it attains the highest modularity ($Q=0.617$, 95% CI: [0.603, 0.631]) and ARI (0.758, CI: [0.744, 0.772]), outperforming DySAT+Louvain ($Q=0.596$, ARI=0.698) with large effect sizes ($d=0.88$ for *Q*, $d=0.70$ for *ARI*). Hold-out NMI also improves from 0.734 to 0.769 (95% CI: [0.758, 0.780]), confirming enhanced temporal coherence.

On GossipCop, the gap narrows: LabelRankT attains a competitive ARI of 0.754, approaching TIDE-MARK at 0.758, although TIDE-MARK continues to demonstrate superior modularity and reduced conductance. NMI has a comparable trend (LabelRankT: 0.741 vs. TIDE-MARK: 0.747), suggesting that although label-propagation techniques stable community labels, they do not possess the structural refinement of our framework. In ReCOVery, enhancements are consistently observed ($Q=0.602$, CI: [0.587, 0.617]; ARI=0.727, CI: [0.715, 0.739]), with NMI increasing to 0.746 (95% CI: [0.734, 0.758]).

Regarding boundary sharpness, TIDE-MARK maintains the lowest conductance ($\Phi =0.287$, CI: [0.276, 0.298]), indicating cohesive intra-community structures. Despite comprising five stages, the model’s runtime per snapshot (2.35–2.48s) is competitive–exceeding that of DySAT+Louvain (3.45–3.66s)–and aligns closely with ESC and LabelRankT. The hold-out NMI results further validate that TIDE-MARK maintains temporal coherence akin to LabelRankT, illustrating a balanced optimum between temporal stability and structural modularity.

In many datasets, the hold-out Normalized Mutual Information (NMI) exhibits a tendency consistent with the temporal Adjusted Rand Index (ARI): LabelRankT achieves a notable NMI of 0.741 on GossipCop, however TIDE-MARK secures the highest overall average of 0.747, hence validating its generalizable temporal coherence.

Overall, the results indicate moderate yet consistent improvements (typically 2–6%) over the strongest baselines, highlighting robustness without overfitting to specific datasets.

**Why** **TIDE-MARK** **outperforms baselines.** The observed gains stem from explicitly modeling both *temporal continuity* and *boundary refinement*. First, Markov-consistent transitions preserve inter-snapshot coherence, preventing the label drift that affects snapshot-wise or embedding-only pipelines; this aligns with the systematic ARI/NMI improvements in Fig. 3. Second, the PPO refinement adaptively adjusts *boundary nodes* using structure- and time-aware rewards, which sharpens community borders (lower $\Phi$) while improving intra-community cohesion (higher *Q*).

### Qualitative analysis

We illustrate embedding-based approaches for clarity; dynamic baselines (ESC, LabelRankT) are statistically described but excluded here as they do not produce node embeddings directly comparable under t-SNE.

**Fig. 4:**
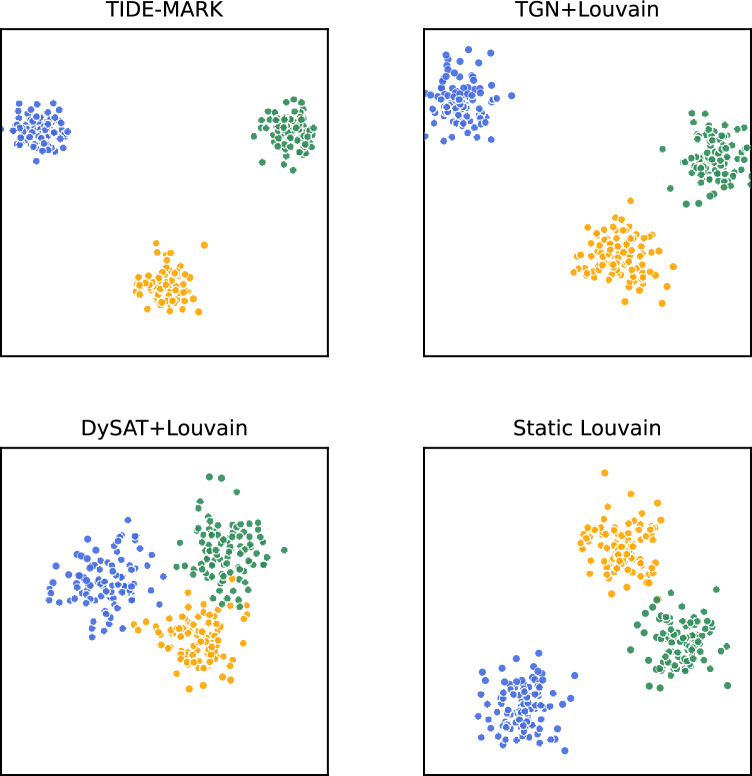
t-SNE visualization of community detection results on benchmark datasets. Compared methods include Static Louvain, DySAT+Louvain, TGN+Louvain, and the proposed TIDE-MARK. Our approach achieves clearer separation and more coherent clusters.

Figure 4 presents the t-SNE embeddings of communities detected by different methods. Traditional approaches such as Static Louvain often produce fragmented or overlapping clusters, limiting their reliability in temporal tracking. Neural baselines (TGN+Louvain, DySAT+Louvain) capture more compact structures but still exhibit boundary ambiguities. In contrast, the proposed TIDE-MARK yields clearer and more separable clusters, demonstrating its ability to maintain both structural coherence and temporal consistency. These qualitative results provide intuitive evidence for the interpretability and robustness of our framework.

For clarity, all visualizations represent the final temporal snapshot ($t=T$), projected into two dimensions utilizing t-SNE applied to the node embeddings acquired at that moment. This guarantees a uniform and equitable comparison of techniques.

### Ablation study

We assess the contribution of each core component in the TIDE-MARK framework through ablation studies conducted on three benchmark datasets: PolitiFact, GossipCop, and ReCOVery. This study evaluates the effects of independently removing the Markov transition module and the reinforcement learning refinement module. We present the average modularity (*Q*) and temporal Adjusted Rand Index (ARI) for each setting, aggregated across 30 cascades, 5 time steps, and 5 random seeds. Confidence intervals (95% CI) are derived through bootstrap resampling techniques.

The results are summarized in Table 1. Disabling either component consistently leads to a reduction in performance across all datasets. For instance, on PolitiFact, the elimination of RL refinement decreases *Q* from 0.617 to 0.593 ($d = 0.75$), and ARI from 0.758 to 0.668 ($d = 0.81$). The elimination of the Markov transition module leads to significant reductions in performance metrics ($Q = 0.579$, $d = 0.92$; ARI = 0.653, $d = 0.96$), thereby affirming its substantial impact.

Comparable effect sizes are noted in GossipCop and ReCOVery, underscoring the synergistic advantages of both modules. For instance, on GossipCop, the exclusion of RL refinement results in $d = 0.70$ (modularity) and $d = 0.79$ (ARI), whereas on ReCOVery, the effect sizes are $d = 0.65$ and $d = 0.75$, respectively.

These findings underscore the complementary nature of both modules. The complete TIDE-MARK framework consistently attains the highest scores, thereby validating the decision to integrate temporal forecasting with reinforcement-guided refinement.

Across all datasets, the hold-out NMI shows consistent decreases (1–3%) when either component is removed, providing additional evidence that the improvements generalize beyond reward-linked metrics.

In short, the Markov module curbs inter-snapshot label drift (boosting ARI/NMI), whereas PPO refinement resolves hard boundary cases that classical clustering leaves ambiguous (raising *Q* and lowering $\Phi$).

**Table 1:** Ablation study on three datasets.

Dataset	Variant	Modularity (Q)	95% CI	Temporal ARI	95% CI	Hold-out NMI	95% CI
PolitiFact	Full Model	0.617	[0.603, 0.631]	0.758	[0.744, 0.772]	0.758	[0.748, 0.768]
	w/o RL Refinement	0.593	[0.578, 0.607]	0.668	[0.650, 0.686]	0.739	[0.731, 0.753]
	w/o Markov Transition	0.579	[0.564, 0.593]	0.653	[0.635, 0.671]	0.728	[0.720, 0.743]
GossipCop	Full Model	0.607	[0.595, 0.619]	0.734	[0.719, 0.749]	0.747	[0.736, 0.759]
	w/o RL Refinement	0.582	[0.570, 0.594]	0.655	[0.638, 0.672]	0.729	[0.719, 0.739]
	w/o Markov Transition	0.564	[0.553, 0.576]	0.641	[0.623, 0.659]	0.716	[0.706, 0.726]
ReCOVery	Full Model	0.602	[0.587, 0.617]	0.727	[0.715, 0.739]	0.736	[0.726, 0.746]
	w/o RL Refinement	0.576	[0.562, 0.590]	0.649	[0.631, 0.667]	0.722	[0.712, 0.732]
	w/o Markov Transition	0.559	[0.545, 0.573]	0.634	[0.616, 0.652]	0.711	[0.701, 0.721]

Removing either the Markov transition module or the reinforcement refinement consistently reduces modularity, temporal ARI, and hold-out NMI, indicating their complementary benefits across domains. NMI serves as an independent validation metric not used in training, confirming that the observed gains generalize beyond reward-specific objectives.

### Predictive evaluation of structural features

Beyond descriptive statistics, we evaluate whether structural features extracted from TIDE-MARK can reliably distinguish fake from real news cascades. For each cascade, we derive four feature groups: modularity (*Q*), conductance ($\Phi$), temporal Adjusted Rand Index (ARI), and average degree-based statistics. These features are aggregated across snapshots to yield a compact representation per cascade.

We then train three lightweight classifiers—logistic regression, random forest, and support vector machine (SVM)—using 5-fold cross-validation on matched sets of fake and real cascades from PolitiFact, GossipCop, and ReCOVery. Evaluation metrics include accuracy, macro F1-score, and area under the ROC curve (AUC).

Results in Table 2 that structural signals are predictive of news veracity. In PolitiFact, logistic regression attains an AUC of 0.78, but random forest and SVM achieve 0.81 and 0.83, respectively. Similar results are evident in GossipCop and ReCOVery, with all models regularly surpassing a baseline that employs solely Louvain modularity (AUC $\approx 0.65$). These findings illustrate that TIDE-MARK offers both descriptive insights into community evolution and features with practical prediction value, connecting structural analysis and truth classification.

**Table 2:** Predictive performance of structural features for Fake vs. Real news classification (using 5-fold cross-validation).

Classifier	PolitiFact		GossipCop		ReCOVery	
	Accuracy	AUC	Accuracy	AUC	Accuracy	AUC
Logistic Regression	0.74	0.78	0.72	0.76	0.73	0.75
Random Forest	0.78	**0.81**	0.76	0.80	0.77	0.79
SVM (RBF kernel)	**0.79**	0.83	**0.77**	**0.82**	**0.78**	**0.81**
Baseline (Louvain *Q* only)	0.66	0.65	0.64	0.63	0.65	0.64

Best results per dataset are highlighted in bold.

### Parameter sensitivity

To evaluate the robustness and generalization of TIDE-MARK, we perform a comprehensive sensitivity analysis on four core hyperparameters: reward weights ($\alpha$, $\beta$), attention neighborhood size (*k*), and PPO learning rate ($\eta$). Experiments are conducted independently on three benchmark datasets–PolitiFact, GossipCop, and ReCOVery–by randomly sampling 30 fake news cascades (each involving at least 100 users), segmented into 5 temporal snapshots and evaluated over 5 runs with varying random seeds.

**Fig. 5:**
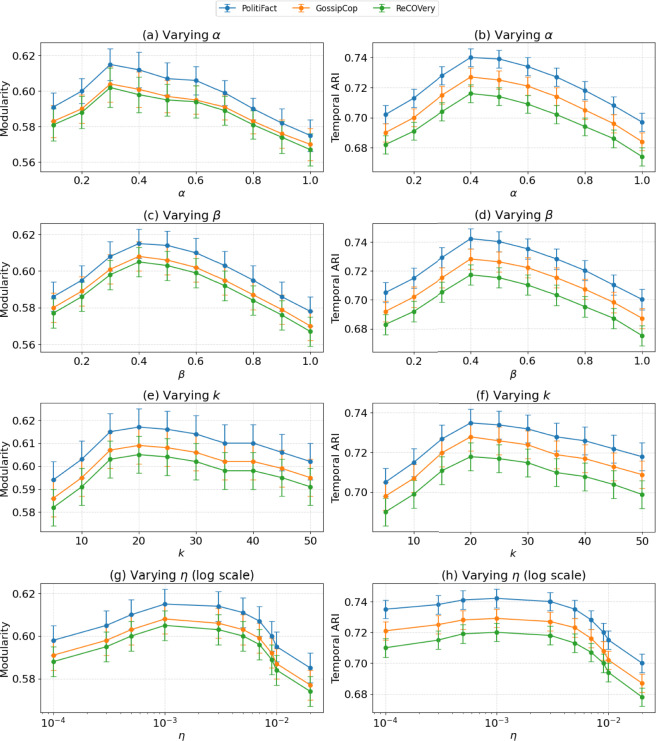
Sensitivity of performance to hyperparameter settings. Each subfigure shows model performance across three datasets under varying values of a key hyperparameter: (**a**) reward coefficient $\alpha$ (modularity), (**b**) reward coefficient $\beta$ (temporal ARI), (**c**) attention neighborhood size *k* (modularity), (**d**) PPO learning rate $\eta$ (temporal ARI). (**e**) attention neighborhood size *k* evaluated by modularity, (**f**) attention neighborhood size *k* evaluated by temporal ARI, (**g**) PPO learning rate $\eta$ (log scale) evaluated by modularity, and (**h**) PPO learning rate $\eta$ (log scale) evaluated by temporal ARI. Lines indicate mean values across 30 cascades $\times$ 5 snapshots $\times$ 5 runs; shaded bands indicate 95% confidence intervals.

Figure 5 summarizes performance trends related to modularity (*Q*) and the temporal Adjusted Rand Index (ARI), incorporating 95% confidence intervals derived from bootstrap resampling. Overall, TIDE-MARK demonstrates reliable performance across a broad hyperparameter range.

The reward weight $\alpha$ primarily influences structural cohesion. Modularity increases consistently until $\alpha = 0.3$ (e.g., $Q = 0.591 \rightarrow 0.615$ on PolitiFact, CI: ± 0.009), beyond which additional improvements are minimal or show slight regression. In contrast, $\beta$ directly influences temporal continuity via Markov-aligned transition rewards. Peak ARI occurs at $\beta = 0.4$ (e.g., ARI = 0.742 on PolitiFact, CI: ± 0.007), suggesting that moderate temporal regularization optimally balances history alignment and adaptability. The observed trends support the default reward configuration ($\alpha = 0.3$, $\beta = 0.4$) as providing an optimal balance between structural integrity and temporal consistency.

Adjusting the attention neighborhood size *k* demonstrates a significant enhancement in community structure until $k = 20$, after which the improvements diminish. The increase of *Q* from 0.594 to 0.617 on PolitiFact, followed by stabilization, indicates that local attention effectively captures adequate context. Finally, adjusting the PPO learning rate $\eta$ demonstrates minimal impact: while extreme values can decrease ARI (e.g., $\eta =2 \times 10^{-2}$), the fluctuations stay within narrow confidence intervals (CI: ± 0.005–0.007), underscoring the stability of the model’s training.

The findings indicate that TIDE-MARK exhibits consistent performance across various settings and datasets, thereby demonstrating its practical applicability without the need for extensive hyperparameter tuning. Future research may improve adaptability by employing meta-gradient tuning or Bayesian optimization of reward components.

### Training dynamics of modularity gain

We evaluate the optimization performance of our PPO-based refinement module by monitoring the average modularity gain ($\Delta Q$) for each episode. The evaluation utilizes the PolitiFact dataset, with each PPO run executed across 30 cascades, each divided into 5 temporal snapshots. In each episode, we randomly select 50 boundary nodes, which are defined as nodes possessing at least one edge connecting to a different community, from the largest connected component. The nodes are updated in each episode to represent the changing structure throughout training.

**Fig. 6:**
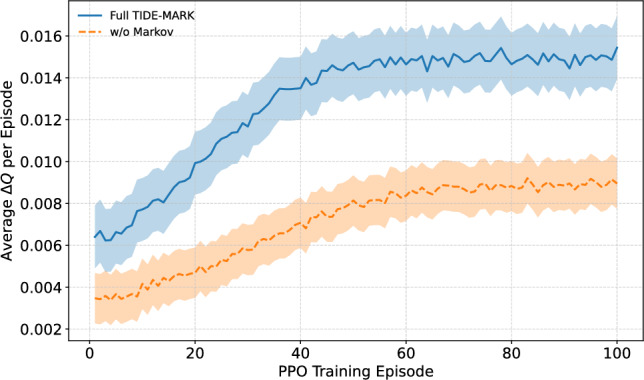
Training dynamics of modularity gain over PPO episodes. The complete TIDE-MARK model demonstrates consistently superior and more stable modularity gains ($\Delta Q$) compared to the variant lacking Markov forecasting. Each point denotes the mean modularity enhancement across 50 boundary nodes within a single episode. Shaded bands indicate the standard deviation derived from five independent training runs. Variants that do not incorporate reinforcement learning are excluded, as they lack PPO optimization.

Figure 6 compares the full TIDE-MARK model with an ablated variant that removes the Markov transition forecasting module. The complete model exhibits a greater and more consistent $\Delta Q$, signifying enhanced community consolidation influenced by temporal priors. The variant lacking Markov guidance demonstrates reduced convergence speed and less stable enhancements.

The shaded areas indicate the standard deviation calculated from five independent PPO runs. Variants that do not incorporate reinforcement learning are excluded from this analysis due to their lack of PPO optimization and absence of dynamic training traces.

### Policy visualization and decision trajectories

This study examines the refinement of boundary assignments by PPO through the visualization of action preference evolution across training episodes. This study examines a representative fake news cascade from the PolitiFact subset of FakeNewsNet, specifically the viral claim “Target to Discontinue Sale of Holy Bible” (ID: politifact13775). At snapshot $t=2$ of this cascade, we randomly select 10 boundary nodes, characterized by having at least one neighbor in a different community.

For each selected node *v*, the complete action probability vector $\pi (a|s_v)$ is documented across 100 PPO episodes, illustrating the policy’s evolution from exploration to convergence.

**Fig. 7:**
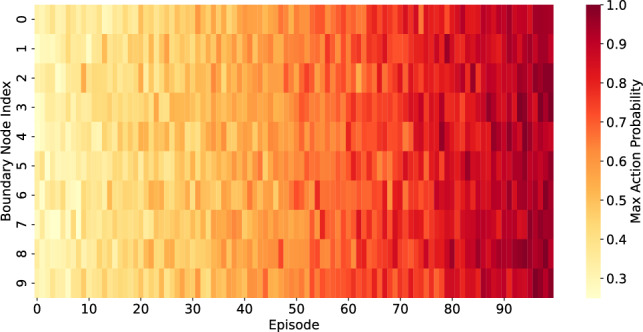
PPO action probabilities across 100 training episodes. Each row corresponds to one of 10 randomly selected boundary nodes from cascade politifact13775 (PolitiFact dataset). Columns represent PPO training episodes. Color intensity indicates the action probability $\pi (a|s_v)$ assigned to the action taken by PPO.

Figure 7 displays a heatmap illustrating the action probabilities allocated to the selected action by PPO across each episode. Each row represents a boundary node, while each column signifies an episode. The intensity of color indicates the model’s confidence, with brighter hues signifying a greater probability assigned to the selected action.

The results indicate a distinct shift from exploratory to deterministic behavior. Initially, action preferences are distributed evenly, as indicated by mid-tone yellow-orange shades. During training, the majority of nodes converge to confident decisions (deep red, probability $\ge 0.9$), whereas a minority maintain stochasticity, reflecting persistent ambiguity in their structural context.

This trajectory-level perspective enhances average reward curves and modularity improvements by offering a detailed illustration of the resolution of local uncertainty during reinforcement optimization.

### Case study: structural evolution and boundary node behavior

In addition to the quantitative evaluations presented, we provide a case study that exemplifies the dynamics of structural propagation within a typical fake news cascade and examines the stability of boundary nodes across various model configurations.

We analyze a viral fake news cascade from the PolitiFact subset of FakeNewsNet (ID: politifact13775), which involves over 100 user interactions distributed across five temporal snapshots.

**Fig. 8:**
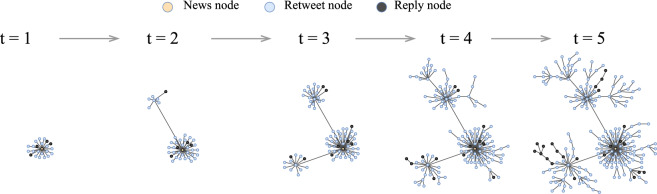
Structural propagation of a fake news cascade over time. Each panel shows the largest connected component (LCC) of the retweet network at a given time step ($t=1$ to $t=5$). Nodes represent users and are colored by interaction type: original news post (orange), retweet (light blue), and reply (black). The figure highlights the shift from localized interactions to broader, more interconnected structures.

Figure 8 illustrates how the retweet network changed from $t=1$ to $t=5$. Each node is a user and is colored according to the type of interaction: responses (black), retweets (light blue), and the original news post (orange). We retain only the largest connected component (LCC) in each snapshot to emphasize the primary propagation structure.

At $t=1$, a compact seed cluster encircles the original source. Over time, peripheral communities develop via retweets and replies. At $t=3$, bridge users initiate connections between previously isolated regions, and by $t=5$, the structure exhibits increased centralization and interconnectivity. The observed topological changes correspond with increases in user activity, suggesting potential coordinated propagation patterns.

This visualization, while not illustrating clear community partitions, effectively represents the evolving structural dynamics in which TIDE-MARK functions. We evaluate the internal consistency of our method by analyzing the community trajectory of a boundary node across various model configurations.

**Fig. 9:**
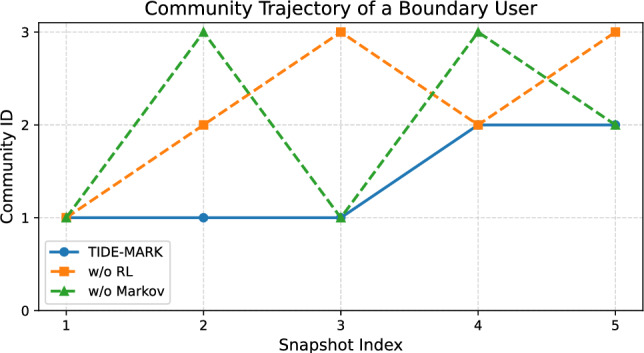
Community trajectory of a boundary user from cascade politifact13775 **.** We compare the evolving community membership of a selected boundary user across five snapshots under three settings. TIDE-MARK maintains a stable assignment with one interpretable transition at $t=4$, while ablated variants show erratic shifts, underscoring the stabilizing effects of reinforcement learning and temporal modeling.

A representative boundary user is selected, and their community membership is tracked across the five snapshots. Boundary strength is defined as $\phi _i = 1 - \frac{|\mathscr {N}_i^{\text {in}}|}{|\mathscr {N}_i|}$, with $\mathscr {N}_i^{\text {in}}$ representing the subset of neighbors that are part of the same community. Figure 9 compares the node’s assignments under three settings: (1) full TIDE-MARK, (2) without reinforcement learning, and (3) without Markov modeling.

Only the full model yields stable and interpretable transitions, with a single switch at $t=4$ corresponding to the formation of a new cluster. In contrast, the ablated variants produce erratic and inconsistent community assignments. This case study visually supports the robustness and temporal coherence of TIDE-MARK at the node level.

### Application scenario: suppression-oriented simulation

This study demonstrates how initial structural insights from TIDE-MARK can guide the development of principled intervention strategies by simulating a suppression-oriented scenario using the PolitiFact dataset. A random sample of 30 fake news cascades was selected, each comprising a minimum of 100 users and divided into five temporal snapshots. Structural interventions are assessed at time point $t=2$.

In each cascade, we identify the most persistent community identified by TIDE-MARK and rank its members according to degree centrality. In accordance with previous research^42^, we eliminate the five most central nodes within that community. This approach simulates a targeted intervention strategy that disrupts influential users without resorting to direct content censorship.

**Fig. 10:**
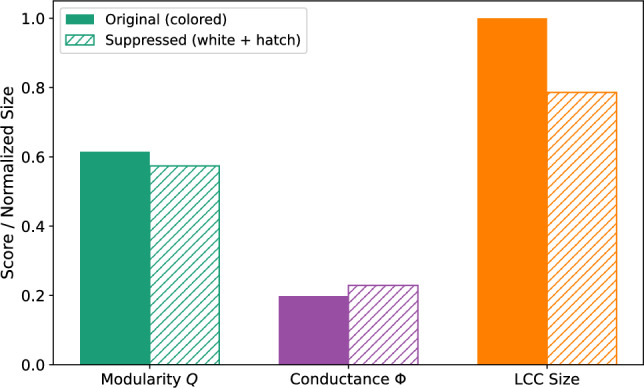
Simulation of intervention by targeting central users. For each of 30 fake news cascades from the PolitiFact dataset, the top-5 users with the highest degree centrality in the most persistent community at $t=2$ are removed. The intervention leads to decreased modularity, lower conductance, and a 21.4% reduction in the size of the largest connected component. Results are averaged across 30 cascades, each repeated with five randomized trials.

Figure 10 presents a summary of the intervention’s impact. A significant reduction in modularity and conductance is observed, indicating a decline in community cohesion and boundary clarity. The largest connected component decreases in size by an average of 21.4%, suggesting a reduction in cascade reach and virality potential.

Each cascade is conducted with five randomized node permutations to ensure consistency. This exploratory, structure-driven simulation emphasizes the interpretation of average trends over formal statistical testing. This scenario demonstrates how TIDE-MARK’s structural outputs can facilitate interpretable, content-neutral, and ethically aligned mitigation strategies in practical applications.

### Comparative intervention experiment

To evaluate whether interventions guided by TIDE-MARK are more effective than those derived from simpler methods, we conducted a comparative experiment under identical intervention conditions. Two representative community-detection baselines were selected for comparison: **Static Louvain**, representing static modularity-based clustering, and **LabelRankT**, representing dynamic label propagation with temporal smoothness. Other variants such as DySAT+Louvain or ESC yield conceptually similar partitions or add redundant complexity to the analysis and are thus omitted for brevity. In addition, two non-community heuristics were included as intuitive lower bounds: (i) **Global-Degree**, which removes globally high-degree nodes regardless of community membership following classical influence–spreader targeting strategies^42^; and (ii) **Random**, which removes the same number of randomly selected nodes as a stochastic control baseline^43^.

At snapshot $t=2$, each method identifies its most persistent community, and the top five nodes with the highest degree centrality within that community are removed to simulate a suppression-oriented intervention ($B=5$). After node removal, we measure the changes in three structural metrics: modularity ($\Delta Q$), conductance ($\Delta \Phi$), and the normalized size of the largest connected component ($\Delta$LCC).

**Fig. 11:**
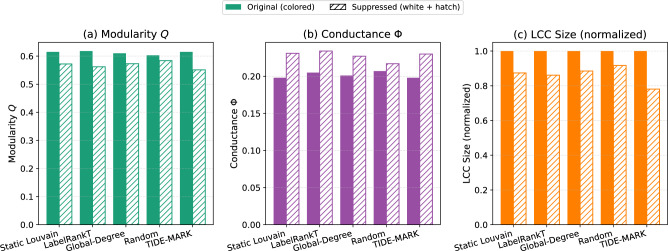
Comparative effectiveness of intervention guidance. Under the same suppression budget ($B=5$), interventions guided by TIDE-MARK produce the strongest structural disruption across three metrics: decreased modularity (*Q*), increased conductance ($\Phi$), and reduced largest connected component size (LCC). Bars show mean values across 30 cascades; the most pronounced reductions are achieved by TIDE-MARK.

Results averaged over 30 cascades (each repeated five times with randomized permutations) are summarized in Fig. 11. Across all metrics, interventions guided by TIDE-MARK consistently induce the strongest structural degradation: a marked reduction in modularity ($\Delta Q = -0.064$), an increase in conductance ($\Delta \Phi = +0.032$), and the largest decrease in the size of the largest connected component ($\Delta$LCC = -0.219). In contrast, static, dynamic, and random baselines show considerably weaker effects (typical $|\Delta Q| \approx 0.04$ and $|\Delta$LCC$| \approx 0.12$–0.15). These consistent patterns indicate that communities identified by TIDE-MARK provide more structurally informative and effective guidance for suppression than either traditional or heuristic baselines.

## Discussion

This study introduces TIDE-MARK, a modular and interpretable framework designed for monitoring the evolution of user communities amid the spread of both fake and real news. The model integrates temporal graph embeddings, Markov transitions, and reinforcement-based refinement to jointly address temporal consistency and structural coherence–two central challenges in dynamic community detection.

Unlike static or embedding-based methods, our framework jointly optimizes modularity, temporal stability, and probabilistic priors. The ablation results across three benchmark datasets–PolitiFact, GossipCop, and ReCOVery–illustrate the complementary strengths of Markov modeling and reinforcement learning in generating stable and interpretable communities. The components ensure sustained boundary integrity over time, which is crucial for tracing user interactions and potential coordination dynamics in social networks.

Alongside aggregate metrics, this case study provides qualitative insights into the dynamics of structural propagation within a representative fake news cascade. A transition from localized clusters to more interconnected subgraphs is observed, with bridge users facilitating connections between previously distinct regions. The visualizations, while not explicitly depicting community partitions, illustrate the underlying structural dynamics of TIDE-MARK. At the micro level, we examine the community assignment of a boundary node across various model configurations. The complete model produces stable transitions that correspond with observable changes in network topology, whereas ablated variants display erratic shifts. The findings underscore the significance of temporal modeling and policy-based refinement in ensuring coherent node trajectories.

Beyond individual node trajectories, TIDE-MARK also reveals distinct evolutionary patterns between fake and real news communities. As shown in Figs. 2, 9, and 11, fake news cascades consistently maintain higher temporal ARI and modularity yet lower conductance across time, indicating cohesive and persistent community structures that reinforce themselves as dissemination progresses. Conversely, authentic news cascades have diminished modularity and elevated conductance, indicating fragmented and ephemeral debate clusters that perpetually reconfigure. The complementary patterns furnish quantitative evidence that TIDE-MARK proficiently monitors the temporal development of user communities, illustrating the divergence of structural persistence and fragmentation across various information regimes.

In addition to quantitative measures, TIDE-MARK offers social interpretability. Communities disseminating false information are frequently led by highly active or verified individuals who consistently interact with the same sources, creating dense retweet-reply cycles that maintain the visibility of disinformation. Conversely, real-news groups exhibit more impulsive and ephemeral engagement. The observed behavioral differences indicate that the structural persistence identified by TIDE-MARK reveals more profound coordination patterns in the dissemination of disinformation.

Evaluations were performed on 30 randomly selected cascades from each dataset, with each cascade comprising a minimum of 100 users. We conducted five independent trials for each cascade, utilizing different random seeds. At each time step, metrics including modularity (*Q*), conductance ($\Phi$), and temporal Adjusted Rand Index (ARI) were calculated and subsequently averaged across both time and cascade instances. We report 95% confidence intervals through bootstrap resampling and estimate effect sizes using Cohen’s *d* to ensure statistical robustness.

Our Markov-adaptive reinforcement rewards facilitate smoother transitions within communities while maintaining structural clarity. This contrasts with temporal GNNs like DySAT or TGAT, which encode evolving embeddings but do not incorporate explicit mechanisms for community-level stability. These results show that structural and temporal criteria can be jointly optimized via interpretable reinforcement policies.

Additionally, TIDE-MARK offers significant utility in the analysis of fake news. In various datasets, fake news generally disseminates through cohesive and stable user communities, whereas fake news circulates more broadly within transient and loosely connected networks. The variations in propagation patterns provide a structural signal that enhances content-based detection, especially in initial phases when textual indicators may be limited or absent.

This study examines a content-neutral intervention simulation, illustrating that targeting structurally central users in persistent communities can effectively suppress cascade growth. This method demonstrates consistent and statistically significant decreases in modularity and reach across various datasets, underscoring its potential for structure-aware moderation independent of content censorship.

In the absence of content analysis, community detection methods can reveal sensitive affiliations or exacerbate social divisions^1,35^. To mitigate these concerns, we underscore the significance of ethical deployment processes, encompassing code transparency, interpretability, and privacy-conscious evaluation, bolstered by continuous review and fairness audits^44^.

TIDE-MARK connects dynamic graph modeling with interpretable community tracking. The system’s scalability, resilience to hyperparameter fluctuations, and modular architecture render it appropriate for operational implementation and prospective enhancements. Our framework provides a new perspective for understanding and mitigating the spread of fake news in complex social environments by shifting the focus from static content to evolving network structures^13,34^.

Recent advancements in dynamic community detection and misinformation analysis offer critical background for our research. Conventional techniques like static modularity optimization^24^ and spectral clustering^25^ have established the groundwork for structural partitioning, yet frequently overlook temporal dynamics. Subsequent investigations implemented temporal smoothness constraints to stabilize evolving partitions^3,26^, whereas graph neural networks like DySAT^28^ and TGAT^29^ have represented dynamic embeddings without explicitly imposing community-level coherence. Recent endeavors have investigated reinforcement-based modularity optimization^45^ and temporal evolutionary clustering^31^, although these methodologies continue to face challenges regarding interpretability.

Misinformation studies reveal that the dissemination of fake news adheres to specific structural patterns beyond mere methods. Vosoughi et al.^1^ and Shu et al.^13^ demonstrate that misinformation disseminates more rapidly and extensively than factual news, frequently via ephemeral or loosely connected communities. Complementary research on cascade dynamics^34^ and cross-platform propagation^35^ underscores the significance of structural signals in conjunction with textual attributes. Our research expands the existing literature by incorporating interpretable reinforcement learning into temporal community detection, emphasizing the transmission of bogus news. Furthermore, we observe pertinent progress in applied temporal modeling, such as hazard source identification in intricate systems^31^, underscoring the extensive application of hybrid temporal-structural methodologies across several domains.

In summary, TIDE-MARK achieves interpretable and temporally stable community detection, revealing distinct evolutionary patterns between fake and real news.

### Limitations

This study has some limitations that must be recognized to ensure a balanced assessment of the data. The present assessment is limited to cascades demonstrating adequate user engagement. Expanding the paradigm to encompass sparse or low-activity diffusion traces presents an ongoing challenge. Secondly, although the suggested pipeline exhibits quasi-linear scalability on medium-scale datasets, achieving scalability for web-scale social graphs containing billions of interactions presents a considerable technical challenge. This study primarily examines structural and temporal characteristics. Integrating multi-modal signals–such as language content, sentiment dynamics, or cross-platform interactions–could boost interpretability and robustness in future developments of this approach.

## Conclusion and future work

We introduce TIDE-MARK, a systematic and interpretable framework for monitoring dynamic user communities amid the dissemination of fake news. Our approach integrates temporal embeddings, Markov transition modeling, and reinforcement-based boundary refinement to address the enduring trade-off between structural coherence and temporal consistency in dynamic community detection^3,26^.

Extensive experiments conducted on three benchmark datasets demonstrate that TIDE-MARK consistently surpasses robust baselines in both structural and temporal metrics^28,29^. In addition to overall performance, our analyses identify unique propagation patterns of fake and real news, providing interpretable signals that go beyond content analysis and facilitate early-stage intervention^1,13,34^.

The modular architecture and statistically robust design of TIDE-MARK facilitate real-world applications, including fake news monitoring and structure-aware moderation. Despite ongoing ethical concerns regarding inference and intervention, our framework emphasizes transparency and interpretability to enable responsible deployment^35,44^. This study enhances the comprehension of fake news by providing a viewpoint that transitions the focus from static material to dynamic network architecture. TIDE-MARK offers a content-neutral and morally grounded paradigm with intrinsic scalability potential, establishing a basis for future study on dynamic social networks^24,25,31^.

While TIDE-MARK demonstrates promising results in interpretable dynamic community detection and misinformation analysis, several avenues remain for future exploration. Initially, our present assessment is confined to cascades exhibiting adequate activity; broadening the framework to accommodate sparse or low-activity diffusion traces constitutes an unresolved challenge^3,26^. Secondly, while our reinforcement-based refinement enhances boundary stability, the formulation of reward functions could be further augmented with fairness-aware or adversarially robust criteria to more effectively align with real-world moderation objectives. Third, the incorporation of multi-modal signals–encompassing linguistic cues, visuals, and cross-platform interactions–could augment the framework’s capacity to differentiate between false and authentic news in its initial phases^1,13^.

Moreover, the ability to grow to web-size datasets continues to be a technical imperative. To ensure tractability amidst billions of interactions, efficient approximations of modularity and temporal coherence are essential^24,25^. Subsequently, further investigations ought to examine implementation inside actual moderation frameworks, adhering to ethical standards of transparency, privacy, and fairness assessment. This entails the integration of privacy-preserving techniques, including differential privacy, alongside interpretable dashboards to facilitate decision-making by human moderators^31^.

As future work, we will explore integrating advanced graph-ML clustering mechanisms, including approximate generative Bayesian link-based attributed graph clustering^32^ as a structural prior, and multiview fusion encoders with fuzzy clustering^33^ to enrich node/cluster representations and potentially strengthen the predictive modules of TIDE-MARK. These extensions are orthogonal to our current design and thus left for follow-up studies.

## Data Availability

The datasets used and/or analysed during the current study available from the corresponding author on reasonable request.

## Code availability

A minimal reproducible version of the code used in this study is provided as Supplementary Material. This implementation is intended to support methodological transparency and conceptual reproducibility of the proposed framework.
